# Reliability of a Screening Method Using Antibiotic Disks to Detect Carbapenemases in Glucose‐Nonfermenting Gram‐Negative Microorganisms From Clinical Samples of a Regional Hospital in Southeastern Spain

**DOI:** 10.1002/jcla.25036

**Published:** 2024-04-15

**Authors:** Itahisa Hernández‐Chico, Enrique Rodríguez‐Guerrero, Manuela Expósito‐Ruiz, José María Navarro‐Marí, José Gutiérrez‐Fernández

**Affiliations:** ^1^ Departmento de Microbiología, Facultad de Medicina Universidad de Granada‐Instituto de Investigación Biosanitaria Granada Spain; ^2^ Departmento de Microbiología Hospital Universitario Virgen de las Nieves‐Instituto de Investigación Biosanitaria Granada Spain; ^3^ Departmento de Estadística, Facultad de Medicina University of Granada‐Instituto de Investigación Biosanitaria Granada Spain

**Keywords:** *Achromobacter xylosoxidans*, *Acinetobacter baumannii*, carbapenemases, nonfermenting gram‐negative bacilli, *Pseudomonas aeruginosa*

## Abstract

**Background:**

Infections by glucose‐nonfermenting gram‐negative bacilli (NFGNB) pose a major public health problem due to multiresistance to beta‐lactam antibiotics, especially plasmid‐borne carbapenemases. Their detection by microbiology laboratories is challenging, and there is a need for easy‐to‐use and reliable diagnostic techniques. Our objective was to evaluate an in‐house screening method to presumptively detect carbapenemases in NFGNB in a simple and clinically useful manner.

**Methods:**

The study included 175 NFGNB isolates from urinary, respiratory, and rectal samples. In a triple assay, isolates were incubated at 37°C for 24 h on three solid‐culture media: MacConkey II Agar, 5% Sheep Blood Columbia Agar and Mueller Hinton II Agar; meropenem (MEM) and cefepime (FEP) disks were employed for screening. Studies were then performed on the inhibition halo diameter, scanning effects, and the appearance of mutant colonies, which were compared with those observed using the colorimetric Neo‐Rapid CARB Kit and immunochromatography (NG5‐Test Carba and K‐Set for OXA‐23). Receiver operating characteristic curves were constructed for these data.

**Results:**

Carbapenemases were expressed by 79/175 (45.1%): 19 *Pseudomonas aeruginosa* and 60 *Acinetobacter baumannii*. Optimal inhibition halo diameter cutoffs to detect this resistance on 5% sheep blood agar were as follows: 6 mm (MEM) and 6.5 mm (FEP) for *P. aeruginosa* (in the absence of scanning effects and mutations) and 10.5 mm (MEM) and 16 mm (FEP) for *A. baumannii* (even in the presence of scanning effects).

**Conclusion:**

The combined utilization of MEM and FEP antibiotic disks in 5% sheep blood agar, measuring their inhibition haloes, offers an effective method to predict the presence of carbapenemases as resistance mechanism in *P. aeruginosa* and *A. baumannii*.

## Graphical abstract

The optimal cutoff points to detect the carbapenemase as a function of inhibition halo diameter in 5% sheep blood agar is 6 mm for meropenem and 6.5 mm for cefepime in *Pseudomonas aeruginosa*, in the absence of scanning effects and mutations. We propose the utilization of 5% sheep blood agar with MEM and FEP disks to screen for clinical isolates of carbapenemase‐producing non‐glucose‐fermenting gram‐negative bacilli within the screening of multiresistant microorganisms.

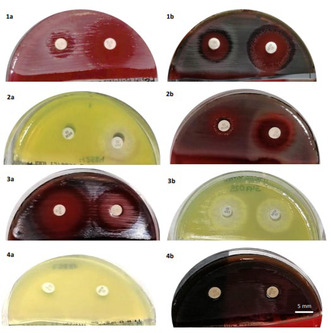

## 1 Introduction

Carbapenemases persist in gram‐negative bacteria almost three decades after their first detection, and their genes have spread throughout most of the world. Knowledge of their prevalence and incidence is important for the implementation of effective preventive and containment strategies. Given the continuing increase in international travel, tourism, and migration, it has become even more necessary to develop guidelines/protocols for the detection and surveillance of carbapenemases at local, national, and global level [1].

Numerous **phenotypic and molecular techniques** of varying effectiveness are available to identify these carbapenemase producers [2, 3]. Among molecular techniques, PCR offers the highest sensitivity (Se) to detect resistance genes, regardless of their activity [4]; however, it is costly and complex, requiring specially trained staff, and it cannot detect new resistance genes [5, 6, 7]. There is a need for less expensive and easier‐to‐use but sensitive techniques to screen for carbapenemase‐resistant pathogens in routine clinical practice [2, 5, 8, 9].

Detection of carbapenemase‐producing isolates is based on a careful susceptibility analysis using automated systems, liquid media, or disk diffusion tests. However, automated systems cannot reliably detect all types of carbapenemase producers, and discrepancies can arise [10]. Previous studies [11, 12, 13] described a cost‐effective and highly sensitive diagnostic method in which the agar antibiogram is simulated by disk‐plate diffusion. This has been proposed as a useful option for the routine diagnosis of ESBL‐ and AmpC‐producing *Enterobacteriaceae*, but further research is required on its value to screen for carbapenemase resistance in glucose nonfermenting gram‐negative bacilli (NFGNB). EUCAST has underscored the importance of detecting carbapenemase‐producing isolates of *Pseudomonas aeruginosa* and *Acinetobacter baumannii* from infection control and public health standpoints [14].

In *P. aeruginosa*, class B carbapenemase metallo‐β‐lactamase (VIM), mainly VIM‐2 is the predominant enzyme in Europe, and KPC producers have also been observed in Latin America [15]. The MBL Etest and disk‐based assays have been used for *P. aeruginosa* over several decades but provide low specificity (Sp) [16, 17, 18, 19], while colorimetric tests have functioned better in *P. aeruginosa* than in *Acinetobacter* [20] and offer the best Sp value described to date. In *A. baumannii*, there have been reports of the presence of type D chromosomal (OXA‐51) and plasmid (OXA‐23, OXA‐24, OXA‐58, OXA 143, and OXA 134) carbapenemases, class A carbapenemases (KPC and GES), and type B carbapenemases (metallo‐β‐lactamases), with the alteration of porins and efflux pumps [21]. Colorimetric tests have generally proven inaccurate in this genus [22]. Genotypic methods should usually be applied to characterize presumptive carbapenemase‐producing strains of *P. aeruginosa* and *Acinetobacter*; however, some of the aforementioned phenotypic techniques may be useful as initial tests, especially in the case of *P. aeruginosa*.

According to the 2023 Guidance on the Treatment of Antimicrobial Resistant Gram‐Negative Infections of the Infectious Diseases Society of America (IDSA) [23], traditional noncarbapenem β‐lactam agents (piperacillin‐tazobactam, ceftazidime, cefepime [FEP], aztreonam) are preferred to treat *P. aeruginosa* isolates, even when these prove susceptible to carbapenems. FEP and both imipenem and meropenem (MEM) were reported to be active against *Acinetobacter* in around half of isolates, although the percentage ranged widely among hospitals [24]. FEP has demonstrated high potency and AmpC stability, its chemical structure is better protected against β‐lactamases [25], and it has been associated with decreased mortality and shorter ICU stay in patients infected with *P. aeruginosa* [26]. Resistance to MEM in *P. aeruginosa* is primarily attributable to the upregulation of efflux pumps or the presence of carbapenemases [27].

Given this background and the widespread rise in carbapenemase‐resistant BGNNF, it appears important to develop effective and easy‐to‐simple approaches to their detection. The present study evaluated a method based on MEM and FEP antibiotic diffusion disks on solid medium, using halo diameter measurements to predict carbapenemase production in NFGNB in a simple manner for possible routine clinical application.

## 2 Methods

### 2.1 Bacterial Isolates

This study included 175 NFGNB prospective isolates (*Stenotrophomonas maltophilia* was excluded as producer of chromosomal carbapenemase) from urine, respiratory system (tracheal and bronchial aspirates), and rectal (swab) consecutive samples, between January 1, 2022, and June 30, 2022, found to exhibit resistance mechanisms by the Microbiology Department of the Virgen de las Nieves University Hospital in Granada (Spain). Isolates were identified by applying the MicroScan system (Beckman Coulter, Brea, CA, USA) and mass spectrometry (MALDI‐TOF, Bruker Daltonik GmbH, Bremen, Germany). Resistance was characterized with the MicroScan system, using current Neg Combo panels and the clinical breakpoints defined by the European Committee on Antimicrobial Susceptibility Testing [28]. Carbapenemases were detected with the colorimetric Neo‐Rapid CARB Kit (Rosco Diagnostica A/S, Taastrup, Denmark) and by immunochromatography (NG5‐Test Carba, NG Biotech, Guipry‐France for KPC, NDM, VIM, IMP, and OXA‐48‐like enzymes and K‐Set, Coris BioConcept, Gembloux, Belgium for OXA‐23) in isolates with values above EUCAST breakpoints for CPB screening. The carbapenemase‐producing type was confirmed by the Andalusian Laboratory of Molecular Typification of the Spanish Program for the Prevention and Control of Healthcare‐related Infections and Appropriate Utilization of Antibiotics (acronym in Spanish, PIRASOA) by massive sequencing (Illumina Inc., San Diego, CA, USA), using CLC Genomics Workbench v10 (Qiagen), ResFinder (Lyngby, Denmark) (https://cge.cbs.dtu.dk/services/ResFinder), and CARD databases (Hamilton, ON, Canada) (https://card.mcmaster.ca/). Most bacterial strains in the present study were adequately characterized by different methods, determining whether they were nosocomial transmitted or transmission clusters, as previously described in this journal [29].

### 2.2 Study of the Effects of FEP and MEM Disks on NFGNB Growth

Each isolate was studied in triplicate on MacConkey II Agar (BD BBLTM, Heidelberg, Germany), 5% Sheep Blood Columbia Agar (BD BBLTM, Heidelberg, Germany), and Mueller Hinton II Agar (BD BBLTM, Heidelberg, Germany). Accordingly, swabs were used to take eight medium‐size punctiform colonies from an initial subculture in UriSelectTM 4 (Bio‐Rad, Francia) of the preserved isolate for mass seeding on the agar, ensuring the uniformity and reproducibility of growth. Half of the culture plate was seeded per microorganism, using the other half for seeding in isolation to verify the purity and to have isolated colonies available for any further study that might prove necessary. After seeding, the 6‐mm FEP (30 μg, Becton Dickinson) and MEM (10 μg, Becton Dickinson) disks were placed equidistantly 30 mm apart and at 15 mm from the plate edge. Media were incubated for 24 h at 37°C. After incubation, the inhibition halo diameter was measured, and the presence of scanning effects and mutations was recorded, describing the latter as scant (S) when solely observed on the outer edge of the inhibition halo or abundant (A) when observed throughout the inhibition halo. Scanning effects refer to observations of bacterial growth with a less homogeneous and translucent cell density than in the rest of the growth on the culture plate surface, forming a partial inhibition halo. In the present context, mutants are small, isolated colonies that appear either on the outer part of the inhibition halo or homogeneously over the whole halo surface. When no inhibition halo was observed, the diameter was recorded as ≤ 6 mm.

### 2.3 Statistical Analysis

The most appropriate culture medium (5% Sheep Blood Columbia, MacConkey II Agar, or Mueller Hinton II Agar) to detect carbapenemase expression was evaluated according to the effects of the antibiotic disks (MEM and FEP). Se and Sp values were calculated for each culture medium and each antibiotic separately, considering that the absence of inhibition halo predicts the presence of carbapenemases. In addition, a receiver operating characteristics (ROC) curve was constructed [30] for each culture medium and each antibiotic separately to determine the halo value in mm with superior Se and Sp for the detection of carbapenemases. Se and Sp values were determined for each cutoff point, and the optimal cutoff in each case was established by calculating the Youden Index (Se + Sp − 1). The association of the scanning effects and the presence of mutations with carbapenemase production was investigated by applying Pearson's chi‐squared test or, when conditions were not met (> 20% of expected frequencies < 5), Fisher's exact test. Likewise, a random sample of the same size (*n* = 6 isolates) was selected from among carbapenemase‐producing *P. aeruginosa* for the study of *Achromobacter xylosoxidans*, although the sample size was too small to determine the diagnostic accuracy of the disks for detecting carbapenemase production. IBM SPSS Statistics 19 (Armonk, NY) and Microsoft Excel 2019 (Redmond, WA) were used for data analyses, considering *p* < 0.05 to be significant in all tests.

### 2.4 Ethics Approval and Consent to Participate

The study protocol was conducted in accordance with the Declaration of Helsinki and the ethical considerations of epidemiological research. This was a noninterventional study, with no further investigation to routine procedures. The biological material was used only for the standard diagnosis of infections as ordered by attending physicians. No additional sampling or modification of the routine diagnostic protocol was performed. Data analyses were performed using a completely anonymous database, where subjects were replaced by different infectious episodes, occurring at least 6 weeks apart from the previous one, if any. Permission to access and use the data was granted by the Clinical Microbiology Management Unit of Virgen de las Nieves University Hospital (Granada, Spain). Ethics committee approval was considered unnecessary according to national guidelines (Law on Data Protection‐Organic Law 15/1999 of December 13 on the protection of data of a personal nature, https://www.boe.es/eli/es/lo/1999/12/13/15).

## 3 Results

### 3.1 *Pseudomonas aeruginosa* (Table 1, Table S1, Figure S1)

**TABLE 1:** Inhibition halo diameters by antibiotic and culture medium in carbapenemase‐producing and nonproducing isolates.

Microorganism	No. of samples	Culture media	Meropenem			Cefepime		
			Halo (mm)	Mean	Standard deviation	Halo (mm)	Mean	Standard deviation
Carbapenemase‐producing isolates								
*P. aeruginosa*	19	5% sheep blood agar	≤ 6	6	0	≤ 6	6	0
	19	Mueller Hinton II Agar	[6–10]	6.26	1.15	≤ 6	6	0
	19	MacConkey II Agar	[6–13]	6.10	2.69	≤ 6	6	0
*A. baumannii*	60	5% sheep blood agar	[6–15]	8.63	2.83	[6–15]	11	2.79
	60	Mueller Hinton II Agar	[6–12]	6.33	1.23	[6–15]	7.71	3.62
	60	MacConkey II Agar	[6–17]	11.56	2.64	[6–20]	14.26	2.59
Noncarbapenemase‐producing isolates								
*P. aeruginosa*	84	5% sheep blood agar	[6–40]^a^	21.24	8.58	[6–40]^a^	19.66	5.16
	84	Mueller Hinton II Agar	[6–40]^a^	20.94	8.67	[6–40]^a^	19.33	5.72
	84	MacConkey II Agar	[6–43]^a^	24.12	8.09	[6–35]^a^	19.62	4.75
*A. baumannii*	6^b^	5% sheep blood agar	[11–25]	19.17	6.42	[12–20]	16.33	3.39
	6^b^	Mueller Hinton II Agar	[6–23]	17.17	6.4	[6–20]	14.67	5.68
	6^b^	MacConkey II Agar	[17–30]	21.67	6.92	[10–25]	18.17	5.64
*A. xylosoxidans*	6	5% sheep blood agar	[15–30]	23.83	7.36	[14–19]	16.5	1.87
	6	Mueller Hinton II Agar	[10–30]^c^	22.5	9.12	[15–20]	16.67	1.86
	6	MacConkey II Agar	[15–30]	25	7.75	[15–17]	16	1.1

*Note:* Diameter ≤ 5 mm = absence of inhibition halo (antibiotic disk).

^a^ Seven isolates with diameter [6–8] mm have porin OprD mutation.

^b^ One isolate of *Acinetobacter pittii*.

^c^ Isolates with diameter ≤ 15 mm show scanning effects.

#### 3.1.1 Carbapenemase‐Producing Isolates

All strains in this group of isolates had a MIC ≥ 16 mg/L for both FEP and MEM. Among the 19/103 (18.4%) carbapenemase‐producing strains of *P. aeruginosa*, 8/19 (42.1%) were IMP‐8, 5/19 (26.3%) IMP‐16, 1/19 (5.3%) IMP‐23, and 5/19 (26.3%) VIM‐1. The mean halo diameter was smallest on 5% sheep blood agar medium, with a value of 6 mm for both MER and FEP.

**FIGURE 1:**
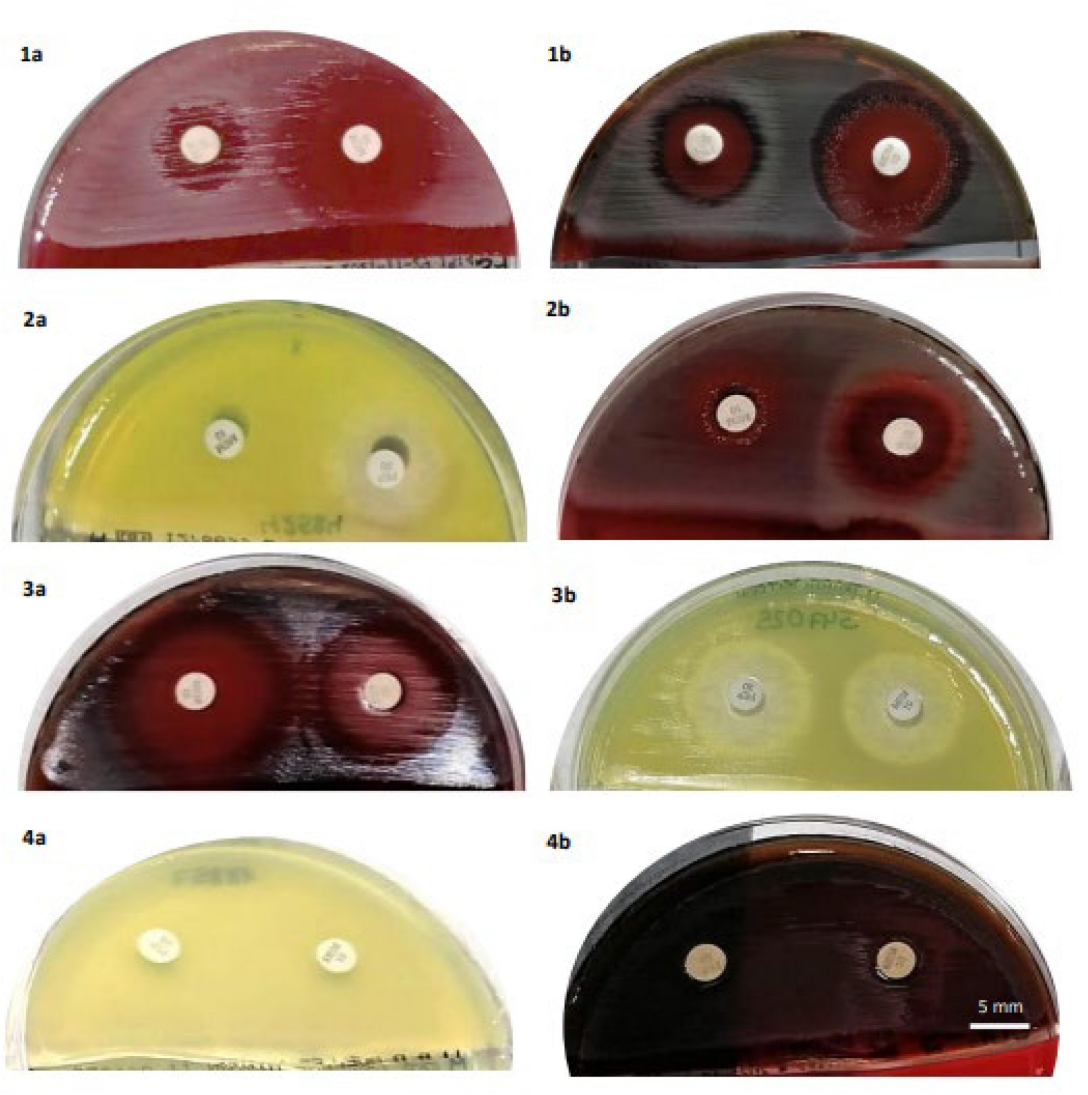
Study of *P. aeruginosa* isolates. Noncarbapenemase producers: 1a. Scanning effect in the inhibition halo of cefepime (FEP) in 5% sheep blood agar. 1b. Scant mutations in the inhibition halo of meropenem (MEM) in 5% sheep blood agar. 2. Appearance of inhibition halos in isolates with porin OprD mutation: a. Absence of halo in MEM in Mueller Hinton II Agar. b. Marked scanning effect with MEM in 5% sheep blood agar. 3. Appearance of noncarbapenemase‐producer isolate with no porin mutation, scanning effect, or mutation: a. 5% sheep blood agar. b. Mueller Hinton II Agar. Carbapenemase producers: 4. Appearance of IMP‐16‐type carbapenemase‐producer isolate with absence of inhibition halo (≤ 6 mm): a. Mueller Hinton II Agar. b. 5% sheep blood agar. 1a, 1b, 2a, 2b, 3a: MEM disk‐left; FEP disk‐right; 3b, 4a, 4b: MEM disk‐right; FEP disk‐left.

Figure 1 and Table 1 exhibit the remaining inhibition halo diameters obtained in carbapenemase‐producing isolates. No isolate showed scanning effects or mutations with either antibiotic.

#### 3.1.2 Noncarbapenemase‐Producing Isolates

All strains in this group of isolates had a MIC ≤ 1 mg/L for both FEP and MEM; 84/103 (81.6%) strains of *P. aeruginosa* were noncarbapenemase producers. The mean halo diameter was smallest on Mueller Hinton II Agar for both MER (20.94 mm) and FEP (19.33 mm).

Figure 1 and Table 1 display the inhibition halo diameters obtained on each culture medium. Halo diameters ≤ 6 mm corresponded to isolates with porin OprD mutation (Table S1).

#### 3.1.3 Selection of Culture Medium for Carbapenemase Detection (Table 2)

**TABLE 2:** Diagnostic accuracy of disks to detect carbapenemase production in *P. aeruginosa* and *A. baumannii*.

Culture media	Antibiotic disks	AUC	CP (mm)	Se (%)	Sp (%)	*J*
*P. aeruginosa*						
5% sheep blood agar	MEM 10	0.964	6	100	92.9	0.929
	FEP 30	0.982	6.5	100	96.4	0.946
MacConkey II Agar	MEM 10	0.975	13.5	100	88.1	0.881
	FEP 30	0.982	6.5	100	96.4	0.964
Mueller Hinton II Agar	MEM 10	0.941	6.5	94.7	89.3	0.840
	FEP 30	0.970	8	100	94	0.940
*A. baumannii*						
5% sheep blood agar	MEM 10	0.972	10.5	85	100	0.850
	FEP 30	0.853	16	100	60	0.600
MacConkey II Agar	MEM 10	0.978	15.5	93.3	100	0.933
	FEP 30	0.725	17.5	91.7	60	0.517
Mueller Hinton II Agar	MEM 10	0.892	14.5	100	80	0.800
	FEP 30	0.807	16	100	60	0.600

Abbreviations: AUC, area under the ROC curve; CP, cutoff point; FEP, cefepime; *J*, Youden Index; MEM, meropenem; Se, sensitivity; Sp, specificity.

In 5% sheep blood agar medium, 100% Se was obtained for both antibiotics, with 92.9% Sp for MEM and 96.4% Sp for FEP. On II Agar, 100% Se was obtained for MEM and 100% for FEP, with Sp values of 88.1 and 96.4%, respectively. On Mueller Hinton II Agar, 94.7% Se was obtained for MEM and 100% for FEP, with Sp of 89.3 and 94%, respectively. Table 2 displays findings obtained for the diagnostic accuracy of the disks to detect carbapenemase production.

### 3.2 *Acinetobacter baumannii* (Table 1, Table S1, Figure S2)

#### 3.2.1 Carbapenemase‐Producing Isolates

All strains in this group of isolates had a MIC ≥ 16 mg/L for both FEP and MEM; 60/66 (91%) of *A. Baumannii* strains were carbapenemase producers, with 54/60 (88.3%) being OXA‐23, 1/60 (1.67%) OXA‐51, and 6/60 (10%) OXA‐58. The mean halo diameter was smallest on Mueller Hinton II Agar for both MEM (6.33 mm) and FEP (7.71 mm).

**FIGURE 2:**
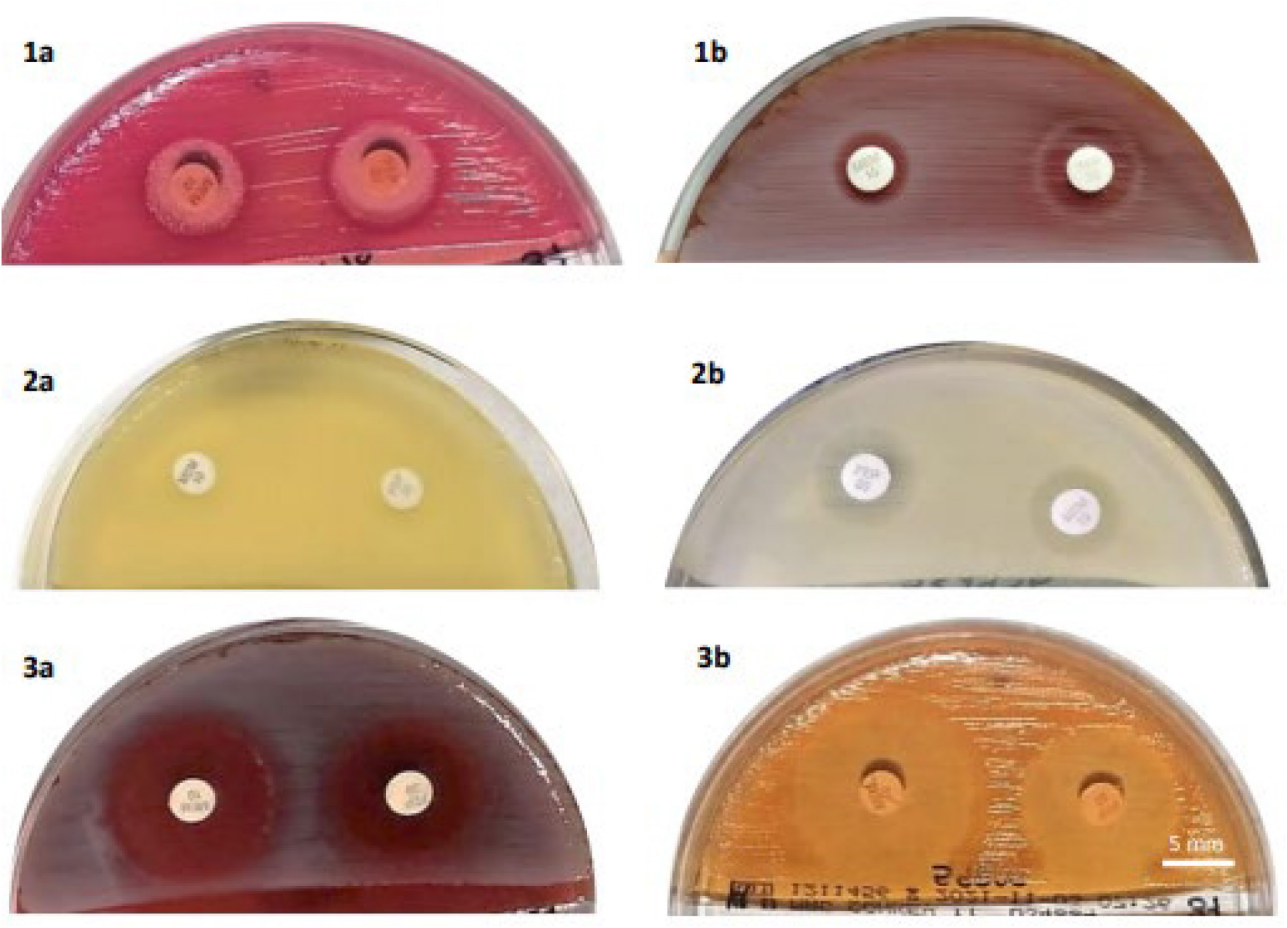
Study of carbapenemase‐producing *A. baumannii* isolates: 1a. Presence of mutations in inhibition halo of MEM on MacConkey II Agar. 1b. Marked scanning effect in inhibition halo of MEM and FEP on 5% sheep blood agar. 2a. Absence of inhibition halos for MEM and FEP on Mueller Hinton II Agar. 2b. Marked scanning effect with MEM and FEP on Mueller Hinton II Agar. Noncarbapenemase producers: 3. Appearance of noncarbapenemase‐producing isolate: a. 5% sheep blood agar. b. MacConkey II Agar. 1a, 1b, 2a, 3a, 3b: MEM disk‐left; FEP disk‐right; 2b: MEM disk‐right; FEP disk‐left.

Figure 2 and Table 1 show the inhibition halo diameters obtained for carbapenemase‐producing isolates. Table S2 exhibits results obtained from the study of scanning effects and mutations. Isolates with inhibition halo had marked scanning effects (Figure 2‐1b), mainly on 5% sheep blood agar medium. However, these same isolates showed a predominant presence of mutations on MacConkey II culture medium (Figure 2‐1a).

#### 3.2.2 Noncarbapenemase‐Producing Isolates

All strains in this group of isolates had a MIC ≤ 1 mg/L for both FEP and MEM; 6/66 (9%) strains of *A. baumanni* were noncarbapenemase producers. The mean halo diameter was smallest on Mueller Hinton II Agar for both MEM (17.17 mm) and FEP (14.67 mm).

Figure 2 and Table 1 display the inhibition halo diameters obtained with noncarbapenemase‐producing *A. baumannii* isolates. Table S2 exhibits findings obtained for scanning effects and mutations.

#### 3.2.3 Selection of Culture Medium for Carbapenemase Detection

Table 2 exhibits the results obtained for the diagnostic accuracy of FEP and MEM disks to detect carbapenemase production. The presence of scanning effects in the FEP inhibition halo on 5% sheep blood agar was correlated with the presence of carbapenemases (*p* = 0.031); however, this association was not observed for the MEM inhibition halo (*p* = 0.350). The presence of mutations in the MEM inhibition halo on MacConkey II Agar was correlated with the presence of carbapenemases (*p* = 0.00129); however, this association was not observed for the FEP inhibition halo (*p* = 1).

### 3.3 *Achromobacter xylosoxidans* (Table 1, Table S1)

**FIGURE 3:**
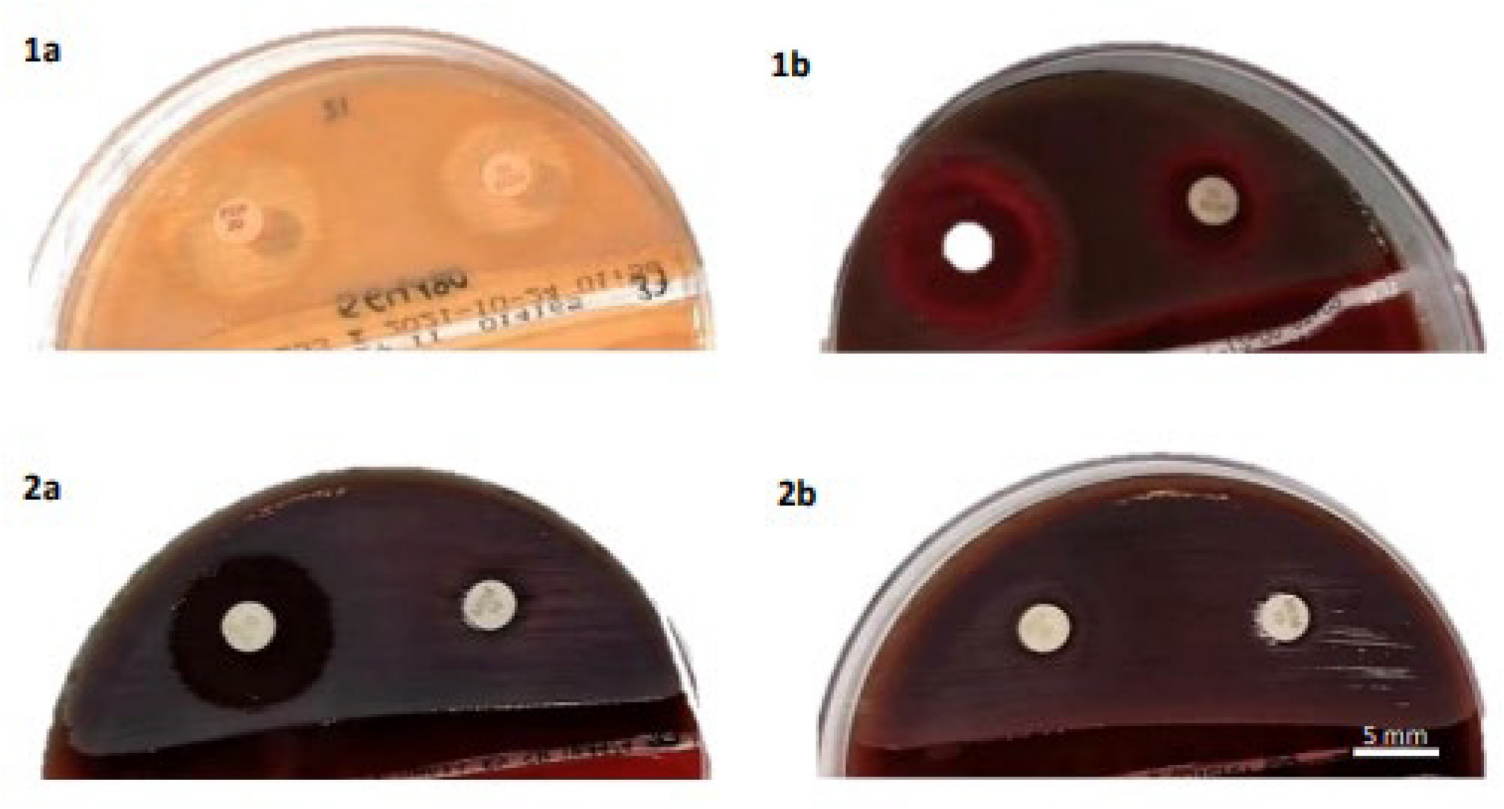
1. Appearance of isolate of noncarbapenemase‐producing *A. xylosoxidans* isolate: a. MacConkey II Agar. b. 5% sheep blood agar. MEM disk‐ right; FEP disk‐left.

All these isolates were noncarbapenemase producers, with a MIC ≤ 1 mg/L for both FEP and MEM. Inhibition halo diameters are displayed in Figure 3 and Table 1. Table S2 reports the results obtained for the presence of scanning effects. No mutations were detected in inhibition halos.

#### 3.3.1 Selection of Culture Medium for Carbapenemase Detection

Se and Sp values were calculated for each antibiotic and culture medium (absence of halo = presence of carbapenemases), obtaining Se and Sp values of 100% for MEM and FEP on all three media under study.

## 4 Discussion

This study included a large sample of carbapenemase‐producing and nonproducing NFGNB obtained from urine, respiratory system (tracheal and bronchial aspirates), and rectal (swab) samples (Table S1). Genetic study of the isolates by the Molecular Typing Laboratory of Andalusia (PIRASOA program) yielded epidemiological information and data on antibiotic susceptibility and on the type of carbapenemase and its clone in carbapenemase‐producing isolates, allowing the detection of occasional horizontal transfer among patients at the hospital. The proposed methodology was also applied, comparing inhibition halos between nonfermenting microorganisms that produce carbapenemases and those that do not. The inhibition halo was always absent (≤ 6 mm) in carbapenemase‐producing microorganisms, whereas its diameter reached up to 40 mm in noncarbapenemase producers, confirming the usefulness and statistical robustness of this methodology, as discussed below.

Phenotypic methods, such as those based on culture and detection media, are easy to use and inexpensive, favoring their routine application in clinical laboratories. However, the Se and Sp values obtained are still not conclusive, and the techniques require long incubation times [31].

Various nonmolecular tests have been proposed over the years for the detection of carbapenemase activity, but none offer 100% Sp and Se [3]. The modified Hodge test (MHT), based on in vivo carbapenemase production, has attracted interest, especially in the USA, but it requires long time periods for Enterobacteriaceae (24–48 h), and its Sp and Se values can be poor [32]. A culture medium containing cefpodoxime, initially designed to detect ESBL producers (ChromID ESBL; bioMerieux, La Balme‐les‐Grottes, France), and a medium containing carbapenem (CHROMagar KPC; CHROMagar, Paris, France) have been evaluated for the detection of carbapenemase producers; however, the former lacks Sp for carbapenemase producers, and both were developed for Enterobacteriaceae [11, 33, 34]. Carba NP and CarbAcineto biochemical tests have also been proposed as relatively fast procedures, but they involve extra costs and are not useful for the routine screening of multidrug‐resistant microorganisms [35, 36].

Lasko et al. [37] evaluated a modification of the method with carbapenemase inactivation (eCIM), increasing the concentration of EDTA, in 24 isolates of *P. aeruginosa* producing IMP and SPM. Their results showed an increase in Se of up to 100%. However, although a promising finding, the number of isolates employed does not allow definitive conclusions to be drawn. de Oliveira et al. [38] recently evaluated the Carba NP, Blue Carba, and mCIM/eCIM methods in *Pseudomonas* spp. with carbapenemases similar to those in our study (KPC, VIM, IMP, and NDM) and reported Se values of 94.7%, 93.6%, and 93.6%, and Sp values of 90.6%, 100%, and 96.8%, respectively. However, the Carba NP test alone was able to differentiate between class A and class B carbapenemases. In addition, only isolates of *Pseudomonas* spp. were studied. A study of KPC‐producing *P. aeruginosa* [39] compared between MHT and phenotypic assays with boronic acid, describing lower Se values (84.6% and 77%, respectively) than achieved with the present method. In another comparative study of phenotypic diagnostic techniques [40], the Se for carbapenemase detection in *P. aeruginosa* was 93%–100% for most assays, and they achieved comparable accuracy rates. However, poor Se values were reported for all the above tests in the identification of carbapenemase‐producing *A. baumannii*.

In contrast, higher values of Se and Sp were obtained for *A. baumannii* with the present method, allowing for a common approach to this species, which is frequently observed in hospitalized patients. *P. aeruginosa* showed the most homogeneous results among the three media, and no scanning effects or mutations were observed in any case. In addition, study of the value of halo absence to predict the presence of carbapenemases obtained 100% Se on 5% sheep blood agar for the two antibiotics, with Sp values of 92.9% for MEM and 96.4% for FEP.

When the simultaneous absence of halo on both MEM and FEP disks was considered to predict the presence of carbapenemases, 100% Se and 92.9% Sp values were obtained, with a cutoff for MEM of 6 mm (absence of halo corresponds to the presence of carbapenemases) and a cutoff for FEP of 6.5 mm, which can be interpreted in the same way. Some isolates that were not carbapenemase producers also lacked an inhibition halo, which might represent a limitation of the methodology; however, the halo was only absent on the MEM disk and never on both disks. PIRASOA analysis revealed that the absence of inhibition halo for some microorganisms was attributable to porin OprD mutations, the main cause of carbapenem resistance in this species [8].

*A. baumannii* species showed a scanning effect in 86.7% of isolates on 5% sheep blood agar medium (Figure 2‐1b) and mutations in 86.7% of isolates on MacConkey II Agar medium (Figure 2‐1a). Application of Fisher's exact test evidenced statistically significant correlations between the presence of scanning effects in the FEP inhibition halo on 5% sheep blood agar and carbapenemase production in the isolate (*p* = 0.031) and between the presence of mutations in the MEM inhibition halo on MacConkey II Agar and carbapenemase production in the isolate (*p* = 0.00129). ROC curve construction (as with *P. aeruginosa*) demonstrated that the diagnostic accuracy was highest using 5% sheep blood agar. When the simultaneous absence of halo on both MEM and FEP disks was considered to predict the presence of carbapenemases, the Se was 85% and Sp was 60%, with a cutoff of 10.5 mm for MEM and 16 mm for FEP, a larger inhibition halo but with a marked scanning effect. A comparative study of different phenotypic methods to detect carbapenemase‐producing *A. baumannii* in a sample of 90 strains described the so‐called optimized carbapenem inactivation method (oCIM) as the best approach, with Se of 92.4% and Sp of 100% [41]. However, unlike the proposed one‐step method, the CIM and its different variants require two steps: incubation of the disk resuspension in liquid medium and incubation of the resulting disk on solid medium.

A previous study in our setting [11] used the ChromID ESBL medium with ertapenem, cefoxitin, and FEP antibiotic disks. In *Pseudomonas* spp., the cutoff for FEP was found to be 18 mm, although Se values were lower than for the present methodology (85.7% vs. 100%) and the sample size was smaller (*n* = 42 isolates). In *A. baumannii* species, however, the cutoff for FEP was 16 mm, in agreement with the present results.

With regard to *A. xylosoxidans*, no mutations were observed and there was a very low presence of scanning effects. The value of halo absence to predict carbapenemases was studied using *P. aeruginosa* isolates, obtaining Se and Sp values of 100% for both MEM and FEP on all three‐culture media. However, statistically significant results could not be obtained for the cutoff points and diagnostic accuracy due to the small sample size (*n* = 6 isolates). Additional research is warranted in a larger number of isolates.

Importantly, the type of carbapenemase cannot be identified by any culture methodology, and patients must be kept in strict isolation until results are obtained (i.e., for at least 48 h and up to 72 h), using molecular techniques to test for carbapenemase activity [2]. This is a limitation, because it does not allow identification of the type of carbapenemase or whether two or more carbapenemases are present. A further limitation of our test is the size of bacterial load required, which can delay the result.

However, we highlight the value of our test for the study of NFGNB *Pseudomonas* and *Acinetobacter* and underscore its simplicity. Currently, all tests have multiple limitations for carbapenemase detection, especially for nonfermenters, posing microbiologists with a major challenge [38]. The main objectives of these phenotypic techniques are to accelerate detection and improve cost‐effectiveness, which are especially useful advantages in the context of outbreaks [2]. The present methodology meets these objectives and offers additional advantages over other techniques. This is because it uses affordable material available in virtually all hospitals and does not require extra specialist staffing, facilitating its application in settings with limited resources.

One limitation in relation to *P. aeruginosa* is that the studied strains (IMP‐8, IMP‐16, IMP‐23) only infrequently produce carbapenemases, and there is a need to validate the study by including commoner carbapenemase‐producing strains (VIM‐2, VIM‐4, KPC). A further limitation is that *A. xylosoxidans* isolates were all noncarbapenemase producers and showed similar behaviors on all three media. These isolates served as negative controls for carbapenemase expression to test the effectiveness of the proposed methodology.

## 5 Conclusion

Carbapenemase‐producing isolates of *P. aeruginosa* lack inhibition halos for MEM and FEP on 5% sheep blood agar (cutoff points of 6 and 6.5 mm, respectively) and show no scanning effects. On the same medium, the presence of scanning effects in the inhibition halo of FEP for *A. baumannii* is significantly correlated with carbapenemase production (cutoff of 10.5 mm for MEM and 16 mm for FEP).

The combined utilization of MEM and FEP antibiotic disks on 5% sheep blood agar, with measurement of their inhibition haloes, offers an effective and statistically robust method to predict the presence of carbapenemases as resistance mechanism in *P. aeruginosa* and *A. baumannii* as part of the routine screening of multidrug‐resistant microorganisms.

## Author Contributions

José Gutiérrez‐Fernández designed the study. Itahisa Hernández‐Chico performed the experiments. Itahisa Hernández‐Chico and José Gutiérrez‐Fernández drafted the manuscript. José María Navarro‐Marí, Enrique Rodríguez‐Guerrero, and José Gutiérrez‐Fernández reviewed and edited the manuscript. Manuela Expósito‐Ruiz carried out the statistical analyses. All authors agreed on the final version.

## Conflicts of Interest

The authors declare no conflicts of interest.

## Supporting information


Data S1.

## Data Availability

The data presented in this study are available in the main text.
