# Supplementary material for: Reliability of a Screening Method Using Antibiotic Disks to Detect Carbapenemases in Glucose‐Nonfermenting Gram‐Negative Microorganisms From Clinical Samples of a Regional Hospital in Southeastern Spain
Source: J Clin Lab Anal. 2024 Apr 15;38(8):e25036. doi: 10.1002/jcla.25036 (PMC11073814; doi:10.1002/jcla.25036)
Supplement: Supplementary file 1 — Data S1. [file JCLA-38-e25036-s001.zip › screening method validation-supplementary tables revisada kike.docx]

**Supplementary Table 1** Resistance mechanisms in the studied microorganisms.

| RESISTANCE MECHANISMS | MICROORGANISMS | NO. SAMPLES | | |
| --- | --- | --- | --- | --- |
| Carbapenemases | *P. aeruginosa* | **(+)^1^:** 19 | | **(-)^2^:** 84 |
|  |  | **Type** | **Clone** |  |
|  |  | **IMP-8:** 8 (42.10%) | **ST348:** 8 |  |
|  |  | **IMP-16:** 5 (26.32%) | **ST253:** 5 |  |
|  |  | **IMP-23:** 1 (5.26%) | **ST175**: 1 |  |
|  |  | **VIM-1:** 5 (26.32%) | **ST179:**3  **ST357:**1  **ST845:**1 |  |
|  | *A. baumannii* | **(+)^1^:** 60 | | **(-)^2^:** 6* |
|  |  | **Type** | **Clone** |  |
|  |  | **OXA-23:**54** (88.33%) | **ST2:** 54 |  |
|  |  | **OXA-51:** 1** (1.66%) | **ST2:** 1 |  |
|  |  | **OXA-58:** 6 (10%) | **ST2:** 6 |  |
|  | *A. xylosoxidans* | **(+):** 0 | | **(-)^2^:** 6 |
| Other mechanisms | *P. aeruinosa* | **Porin OprD alteration:** 7 | | |

+: positive carbapenemase expression -: absence of carbapenemases

* One clinical isolate belonged to the species *Acinetobacter pittii*

**One isolate simultaneously expressed OXA-23 and OXA-51 enzymes

^1^ FEP MIC: > 16 mg/L; MEM MIC: > 16 mg/L by MicroScan system (Beckman Coulter, Brea, CA)

^2^ FEP MIC: ≤ 1 mg/L; MEM MIC: ≤ 1 mg/L by MicroScan system.

^3^ One isolate with FEP MIC of 4 mg/L for by MicroScan system.

**Supplementary Table 2** Presence of scanning effects or mutations in carbapenemase-producing and non-producing isolates.

| Carbapenemase-producing isolates | | | |
| --- | --- | --- | --- |
| *A.baumannii* (n=60) | **5% blood agar** | Scanning effects | Mutations |
|  | MEM 10 µg | 0 | 0 |
|  | FEP 30 µg | 13 | **S:** 1 |
|  | MEM 10 µg + FEP 30 µg | 39 | 0 |
|  | No. samples | 52 (86.7%) | 1 (1.67%) |
|  | **McConkey II** |  |  |
|  | MEM 10 µg | 3 | **A:** 7 **S:** 36 |
|  | FEP 30 µg | 0 | 0 |
|  | MEM 10 µg + FEP 30 µg | 1 | **S:** 9 **A:**3* |
|  | No. samples | 4 (6.67%) | 52 (86.7%) |
|  | **Mueller Hinton II** |  |  |
|  | MEM 10 µg | 1 | 0 |
|  | FEP 30 µg | 0 | **S:** 1 |
|  | MEM 10 µg + FEP 30 µg | 3 | **S:** 1 |
|  | No. samples | 4 (6.67%) | 2 (3.33%) |
| Non-carbapenemase-producing isolates | | | |
| *P.aeruginosa* (n=84) | **5% blood agar** |  |  |
|  | MEM 10 µg | 2** | **A:** 5 **S:** 26 |
|  | FEP 30 µg | 10 | **S:** 1 |
|  | MEM 10 µg + FEP 30 µg | 0 | **A:** 2 |
|  | No. samples | 12 (14.29%) | 34 (40.48%) |
|  | **McConkey II** |  |  |
|  | MEM 10 µg | 0 | **S:** 21 |
|  | FEP 30 µg | 0 | 0 |
|  | MEM 10 µg + FEP 30 µg | 0 | **S:** 3 |
|  | No. samples | 0 (0%) | 24 (28.57%) |
|  | **Mueller Hinton II** |  |  |
|  | MEM 10 µg | 1 | **S:** 19 **A:** 3 |
|  | FEP 30 µg | 6 | 0 |
|  | MEM 10 µg + FEP 30 µg | 0 | **A:** 1 **S:** 1 |
|  | No. samples | 7 (8.33%) | 24 (28.57%) |
| *A. baumannii* (n=6) | **5% blood agar** |  |  |
|  | MEM 10 µg | 0 | 0 |
|  | FEP 30 µg | 0 | 0 |
|  | MEM 10 µg + FEP 30 µg | 2 | 0 |
|  | No. samples | 2 (33.33%) | 0 (0%) |
|  | **McConkey II** |  |  |
|  | MEM 10 µg | 0 | **A:** 1 |
|  | FEP 30 µg | 0 | 0 |
|  | MEM 10 µg + FEP 30 µg | 1 | 0 |
|  | No. samples | 1 (16.66%) | 1 (16.66%) |
|  | **Mueller Hinton II** |  |  |
|  | MEM 10 µg | 0 | 0 |
|  | FEP 30 µg | 0 | 0 |
|  | MEM 10 µg + FEP 30 µg | 1 | 0 |
|  | No. samples | 1 (16.66%) | 0 (0%) |
| *A. xylosoxidans* (n=6) | **5% blood agar** |  |  |
|  | MEM 10 µg | 0 | 0 |
|  | FEP 30 µg | 0 | 0 |
|  | MEM 10 µg + FEP 30 µg | 2 | 0 |
|  | No. samples | 2 (33.33%) | 0 (0%) |
|  | **McConkey II** |  |  |
|  | MEM 10 µg | 0 | 0 |
|  | FEP 30 µg | 0 | 0 |
|  | MEM 10 µg + FEP 30 µg | 0 | 0 |
|  | No. samples | 0 (0%) | 0(0%) |
|  | **Mueller Hinton II** |  |  |
|  | MEM 10 µg | 0 | 0 |
|  | FEP 30 µg | 0 | 0 |
|  | MEM 10 µg + FEP 30 µg | 2 | 0 |
|  | No. samples | 2 (33.33%) | 0 (0%) |

(S-scant/A-abundant) in the inhibition halo of meropenem (MEM) and/or cefepime (FEP)

*3 isolates with A in MEM and S in FEP.

** The 2 isolates with scanning effects in MEM have porin OprD mutation.
