# Supplementary material for: Reliability of a Screening Method Using Antibiotic Disks to Detect Carbapenemases in Glucose‐Nonfermenting Gram‐Negative Microorganisms From Clinical Samples of a Regional Hospital in Southeastern Spain
Source: J Clin Lab Anal. 2024 Apr 15;38(8):e25036. doi: 10.1002/jcla.25036 (PMC11073814; doi:10.1002/jcla.25036)
Supplement: Supplementary file 1 — Data S1. [file JCLA-38-e25036-s001.zip › Supplementary Figures.docx]

**Figure S1. ROC curves for *P. aeruginosa***

| 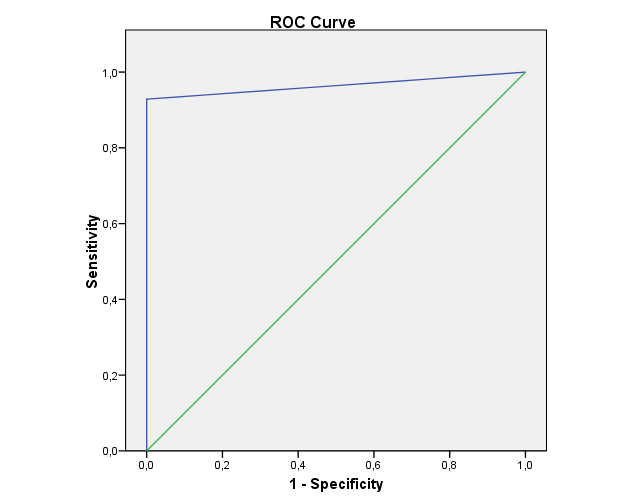  1.a AUC 0.964 | 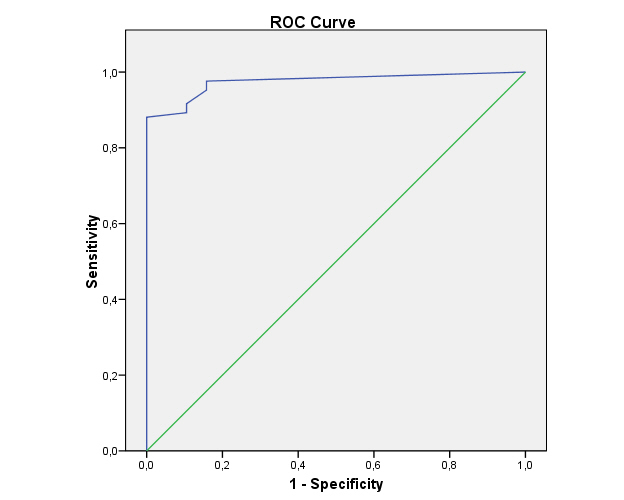  1.b AUC 0.975 | 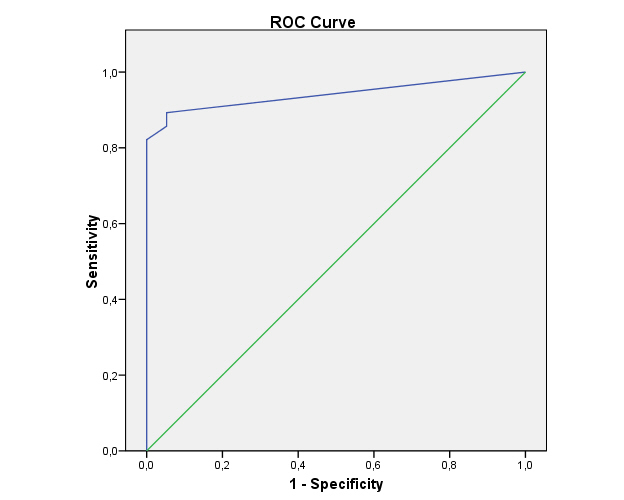  1.c AUC 0.941 |
| --- | --- | --- |
| 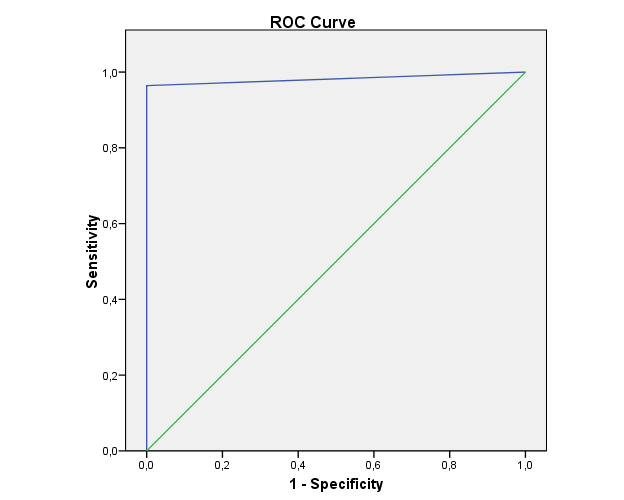  1.d AUC 0.982 | 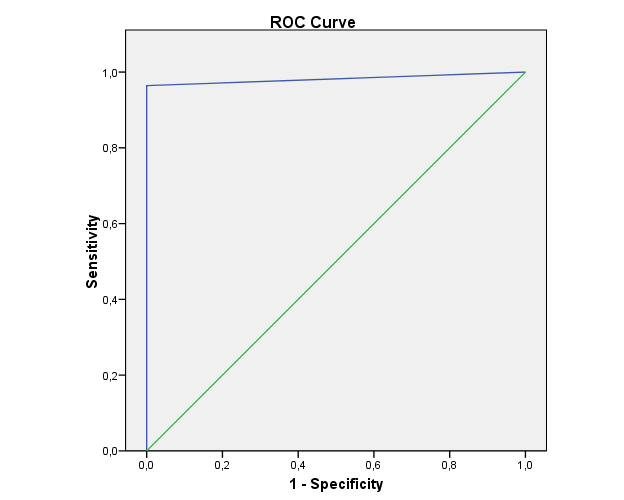  1.e AUC 0.982 | 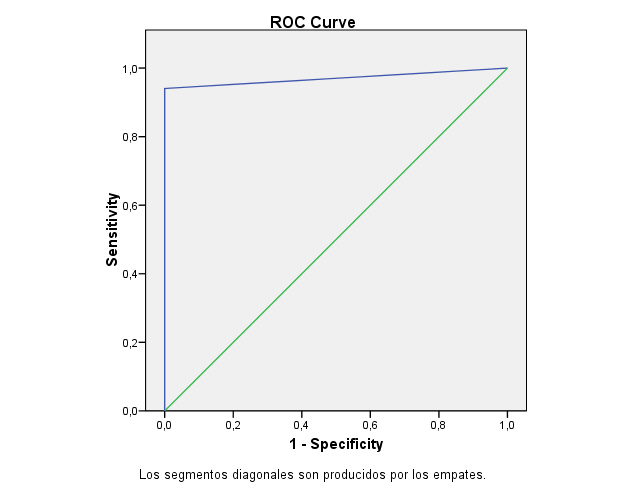  1.f AUC 0.970 |

ROC: receiver operating characteristics curve; AUC: area under the ROC curve. ROC Curve for Antibiotic disk MEM10 on 5% blood agar (1a), MacConkey II agar (1b), and Mueller Hinton II agar (1c). ROC Curve for antibiotic disk FEP 30 on 5% blood agar (1d), MacConkey II agar (1e), and Mueller Hinton II agar (1f).

**Figure S2. ROC curves for *A. baumannii***

| 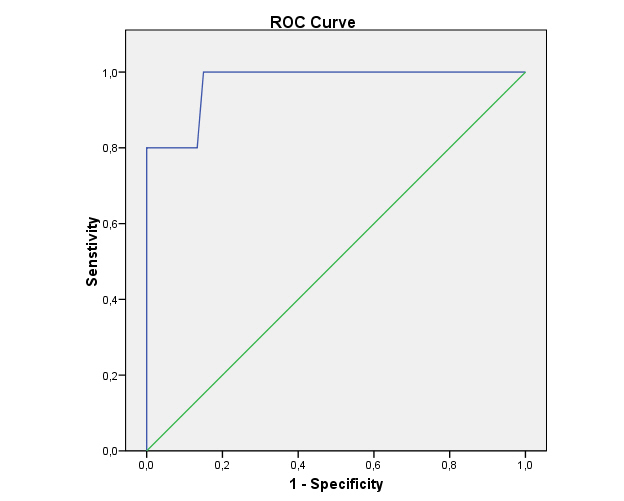  2.a AUC 0.972 | 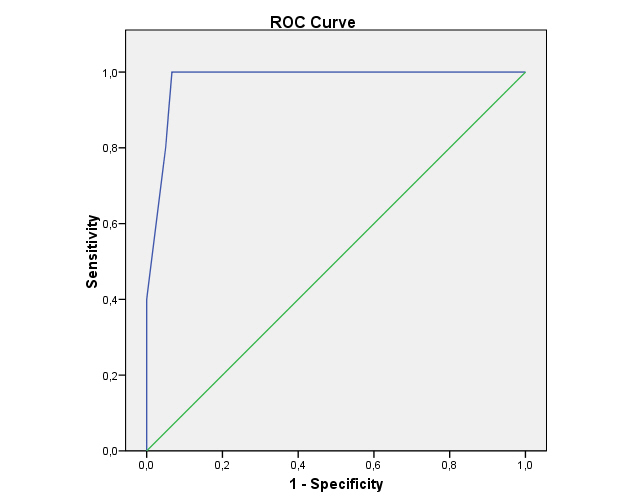  2.b AUC 0.978 | 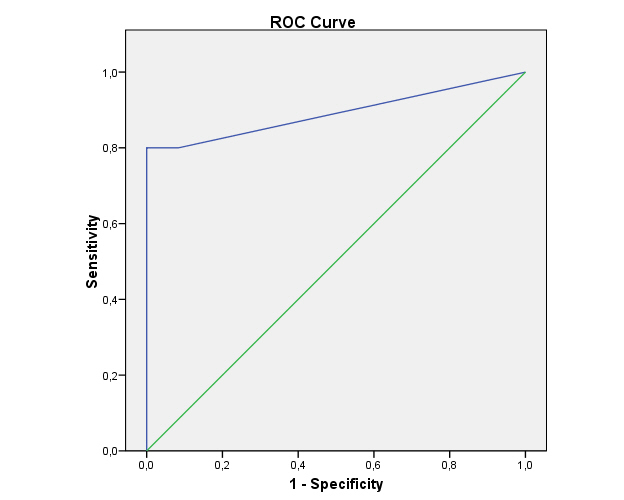  2.c AUC 0.892 |
| --- | --- | --- |
| 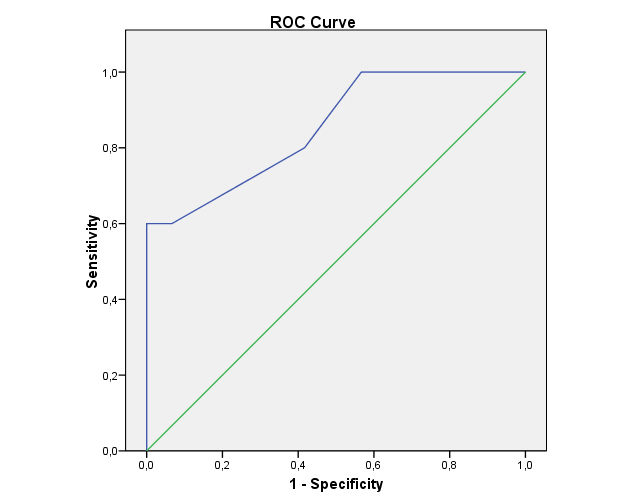  2.d AUC 0.853 | 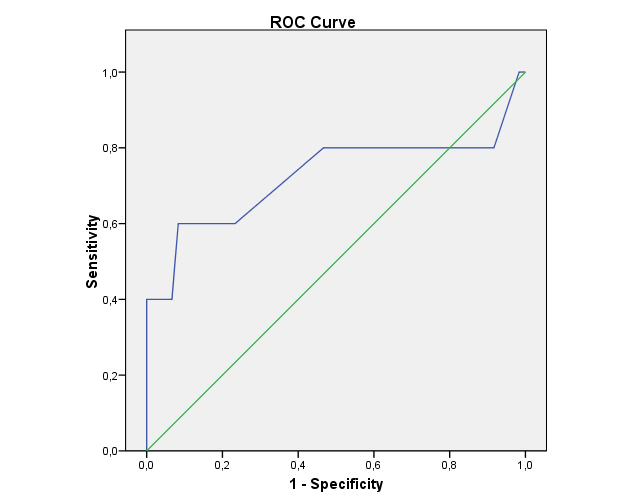  2.e AUC 0.725 | 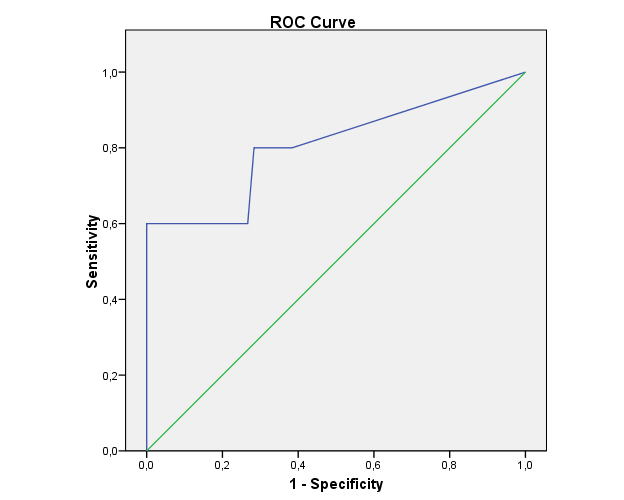  2.f AUC 0.807 |

ROC: receiver operating characteristic curve; AUC: area under the ROC curve. ROC Curve for antibiotic disk MEM10 on 5% blood agar (2.a), MacConkey II agar (2.b), and Mueller Hinton II agar (2.c). ROC Curve for antibiotic disk FEP 30 on 5% blood agar (1.d), MacConkey II agar (1.e), and Mueller Hinton II agar (1.f).
